# Better Dietary Knowledge and Socioeconomic Status (SES), Better Body Mass Index? Evidence from China—An Unconditional Quantile Regression Approach

**DOI:** 10.3390/nu12041197

**Published:** 2020-04-24

**Authors:** Jie Yu, Xiao Han, Hongxing Wen, Jinzheng Ren, Lihong Qi

**Affiliations:** 1 College of Economics and Management, China Agricultural University, Beijing 100083, China; yujie6611@163.com; 2 Agricultural Trade Promotion Center, Ministry of Agriculture and Rural Affairs of P.R. China, Beijing 100025, China; johnjoy@126.com; 3 National Economics Research Center, Guangdong University of Finance and Economics, Guangzhou 510320, China; hxwen2011@126.com; 4 Department of Public Health Sciences, School of Medicine, University of California Davis, Davis, CA 95616, USA

**Keywords:** dietary knowledge, socioeconomic status, body mass index, unconditional quantile regressions

## Abstract

Obesity is a rapidly growing public health threat in China. Improvement of dietary knowledge may potentially reduce the risk of obesity and being overweight. However, existing studies focus on measuring the mean effects of nutrition knowledge on body mass index (BMI). There is a lack of literature on the effect of dietary knowledge on BMI, and the potential heterogeneity of the effect across the whole BMI distribution and across socioeconomic status (SES) groups. This study aims to investigate the heterogeneous nature of the relationship between dietary knowledge, SES, and BMI, using data from the China Health and Nutrition Survey (CHNS) in 2015. We employed unconditional quantile regression (UQR) to assess how the relationship between dietary knowledge, SES, and BMI varies across the whole BMI distribution, and conducted subgroup analyses using different socio-economic subsamples. Results indicate that dietary knowledge had no statistically significant impact on BMI across the BMI distribution. There was a large degree of heterogeneity in the SES effect across the BMI distribution as well as a major gender difference in the SES effect on BMI. Education had a significant and inverse association with BMI across the BMI distribution, greater at higher BMI quantiles. Income growth had a larger effect on the 50th quantile of BMI for males in the middle-income group, but was not significant for females. As income increased, males without college educations had higher BMI while females with college or higher education generally had lower BMI. The findings of this study reveal the heterogeneous nature of the relationship between SES, gender, and obesity across the entire BMI distribution, suggesting that quantile regressions might offer a valuable framework for exploring the complex relationship of dietary knowledge, demographic, and socio-economic factors on obesity.

## 1. Introduction

Obesity is a major public health problem and the fifth leading cause of death globally [1]. Worldwide obesity has nearly tripled since 1975, with 39% overweight adults in 2016 [2]. Overweight and obese people show potentially unhealthy changes in metabolism and, thus, have a high risk of suffering from various chronic diseases. This may impose a heavy burden on healthcare and cause serious social and economic consequences [3,4]. The indirect economic burden caused by obesity is expected to account for 8.7% of GDP by 2025 [5]. To date, the obesity epidemic is an unresolved public health crisis, increasing disease burdens and healthcare costs across the globe.

China had the largest obese population in the world in 2014 [6]. The prevalence of obesity and being overweight showed an accelerating trend nationally [6]. The National Health Commission of China estimated that the prevalence of obesity and being overweight increased from 22.85% to 30.15% and 7.1% to 11.9% from 2002 to 2015, respectively [7]. The obesity crisis may generate long term distress and disabilities, impose a heavy burden on the social security system and potentially constitute a threat to the long-term economic development of China [3]. In response to the obesity threat, the Chinese Government has implemented health education programs and published Dietary Guidelines [8], aiming to improve people’s dietary knowledge, develop better food habits, and maintain healthy lifestyles.

It is believed that nutrition knowledge is one of the important factors for healthy diet [9]. According to the knowledge, attitude, and practice (KAP) model, rich nutrition knowledge may lead to changes in attitude about diet, influence food choices, dietary quality and, consequently, lead to lower risk for obesity [10,11]. However, it was argued that nutrition knowledge might be a necessary but not sufficient factor for changes in consumer food behaviors, suggesting that nutrition knowledge alone may not necessarily have significance influence on obesity risk [12].

Previous research exploring the relationship between nutrition knowledge and body mass index (BMI) showed mixed results. Some studies done among Belgian women, urban men in France, children in Germany, adolescents in the US, and urban adults in Northern Ireland did not demonstrate significant association between nutrition knowledge and BMI [13,14,15,16,17]. However, other studies identified a significant relationship for low-income reproductive-age women in United States, and for Mexican women with a low socioeconomic status (SES), respectively [16,18]. The inconsistent results indicate that the relationship between nutrition knowledge and BMI may depend on socio-demographic characteristics of the study participants.

Existing studies primarily focus on the average effect of nutrition knowledge on adult BMI, but do not investigate possible non-linear relationships or heterogenous effects of nutrition knowledge on obesity across the distribution of BMI, or across subgroups defined by certain demographic and socioeconomic characteristics, such as gender, income, and education. Gender-specific associations between education and income in relation to obesity have been reported [19]. Income may be significantly associated with higher BMI in males, while education may have a significant association with lower BMI in females [20,21]. These results suggest that subgroup effects may exist in the relationship of nutrition knowledge, SES, demographic factors and BMI.

Although nutrition knowledge and dietary knowledge both emphasize the importance of healthy eating, the former focuses on detailed nutrient intake and its linkage to diseases, while the latter stresses the importance of balancing diet and exercise [22]. Studies on whether dietary knowledge is associated with BMI in adults are limited [23,24]. In this paper, we investigate the heterogeneous nature of the relationship between dietary knowledge, SES, and BMI among Chinese adults, using data from the China Health and Nutrition Survey (CHNS) in 2015 [25]. We aim to assess how the relationship between dietary knowledge, SES, and BMI varies across the whole BMI distribution, and across different socioeconomic subgroups, by employing unconditional quantile regression (UQR). To our knowledge, there is a lack of literature on the relationship between dietary knowledge, social status, and BMI in China. This study will address the current gap in the literature by exploring the impact of dietary knowledge and SES on obesity and being overweight in China, using cross-sectional data.

## 2. Materials and Methods

### 2.1. Data and Variables

#### 2.1.1. Data

In this study, we used the data from the China Health and Nutrition Survey (CHNS), designed to examine the effects of health policies and programs on the health and nutritional status of the Chinese population [26]. This ongoing project offers a multipurpose, longitudinal, and household-based survey established as a joint effort between the University of North Carolina at Chapel Hill and the China Center for Disease Control and Prevention. It collects information on nutritional and health status as well as demographic and socioeconomic data of the Chinese population [26]. The first survey was conducted in 1989, and then in 1991, 1993, 1997, 2000, 2004, 2006, 2009, 2011, and 2015. The 2015 CHNS survey implemented a multistage random cluster design in 15 provinces and megacities (i.e., Heilongjiang, Liaoning, Jiangsu, Shandong, Henan, Hubei, Hunan, Guangxi, Guizhou, Beijing, Shanghai, Chongqing, Yunnan, Shanxi, and Zhejiang), including 20,914 individuals from 7319 households. The CHNS raw data and detailed information on survey contents and quality control procedures can be found in the official website (cpc.unc.edu/projects/china). In this study, we used the latest data collected in the 2015 survey to study the effects of dietary knowledge and SES on BMI.

#### 2.1.2. Variables

Dependent variable: Obesity is defined as the excessive accumulation of body fat, and can be measured by different indexes such as waist-to-hip ratio (WHR) and BMI. The BMI is defined as the weight in kilograms divided by the square of the height in meters (kg/m^2^). It is adopted by the World Health Organization (WHO) to classify being overweight and obesity. BMI provides the most useful population-level measure of obesity and being overweight; by far it is also the most commonly used method for determining obesity [27]. BMI was employed as the dependent (effect) variable in our investigation.

Dietary knowledge: We summarized the dietary knowledge based on 18 diet-related questions in the 2015 CHNS survey (Table 1). In each question, respondents were asked to select “strongly disagree”, “disagree”, “neutral”, “agree”, and “strongly agree”. For each answer, we generated a score ranging from −1 to 1, indicating its level of agreement with the correct answer (“true/false” column in Table 1): a score 1 for “agree” and “strongly agree”, 0 for “neutral” or “unknown”, and −1 for “strongly disagree” and “disagree” [28]. We constructed a summary dietary knowledge score by summing up the scores of the 18 questions and then added 10 so that all values were positive, which would not affect the interpretation of the results [29,30]. The maximum dietary knowledge score was 28. A higher score indicates better dietary knowledge.

The validity and reliability of this diet-related questionnaire has been previously evaluated [31,32,33]. We also implemented the Cronbach’s alpha test to examine the questionnaire’s internal reliability. The standard Cronbach’s Coefficient α of the questionnaire was 0.78, greater than the commonly accepted standard 0.7, showing that the questionnaire has relatively high internal reliability.

**Covariates:** Based on the literature, we considered the following commonly used SES and demographic (DE) factors: income, education, age, gender, household size, marital status, and regional variables [19,31,34,35,36,37]. Because the relationship between BMI and household income varies with gender, we constructed new variables using household income per capita in concert with gender, denoted by Male*Household Income Per Capita (PCHHincome) and Female* PCHHincome [38]. Based on the respondents’ answers to the questions about smoking and alcohol intake, we defined two dummy variables to indicate their smoking status (denoted by Smoke: Yes, No) and alcohol intake (denoted by Alcohol: Yes, No), respectively.

Descriptive statistics were generated for the dependent and independent variables of the study respondents and presented in Table 2.

The whole study sample included 9901 respondents, after excluding observations with missing values and respondents who were 18 years old and younger. To examine the relationship between dietary knowledge, SES, and BMI among Chinese adults, unconditional quantile regression (UQR) models were used, where BMI was the dependent (or effect) variable and dietary knowledge, and SES were the independent (or explanatory) variables [25]. All models included gender, age, and age squared, marital status (married or unmarried), male and female household income per capita (Male*PCHHincome and Female* PCHHincome in Equation (4), respectively), education level, smoking status (Smoke) and alcohol intake (Alcohol), household size (HHsize), rural area (rural or urban), and the fixed-effect for each region (*µ*) as well to control for the unobservable regional factors.

Quantile regression, including UQR and conditional quantile regression (CQR), has a fundamental advantage over the ordinary least squares (OLS) estimation [20,29], which can estimate the effects of the independent variables on the different percentiles, i.e., the entire distribution of the dependent variable, not only its mean. When a model is characterized by a set of independent variables, UQR is a more appropriate estimation technique as conditional and unconditional relationships are not necessarily equivalent and may differ significantly [29]. Moreover, the estimate from UQR for a specific independent variable, e.g., dietary knowledge, does not depend on the set of independent variables used in the model, a major advantage of UQR over CQR. It is also robust toward outliers as CQR [29,39].

UQR is based on extending the concept of the influence function to what has been termed the recent influence function (RIF), defined as follows:

(1) $$RIF(y;q_{\tau })=q_{\tau }+\frac{\tau -I\left[y\leq q_{\tau }\right]}{f_{y}(q_{\tau })}$$

where $q_{r}$ is the value of percentile r,$\;f_{\tau }(q_{\tau })$ is the sample density function in the sample percentile τ, and I is a dichotomous variable with value 1 when the value y is less than the corresponding percentile.

After recalculating the variables of interest, Equation (2) can be estimated by the OLS:

(2) $$RIF\left(y;q_{\tau }\right)=X\beta^{UQR}+\varepsilon .$$

An expression illustrating BMI as a function of a set of independent variables can be written as follows:

(3) $$BMI_{i}=f\left(DK_{i,}SES_{i,}DE_{i,}R|\beta \right)+e_{i}$$

where *R* denotes the fixed effects capturing the unobservable factors that may affect the BMI, *DK_i_*, *SES_i_,* and *DE_i_* denote dietary knowledge, SES and demographic factors for the *i*th respondent, respectively, β is the conformable vector of the coefficients to be estimated, and $e_{i}$ is an error term.

The linear functional form of Equation (3) used in our data analyses can be written as follows:

(4) $$\begin{array}{cc} BMI_{i}=\beta_{0}+\beta_{DK} & DK_{i}+\beta_{Age}Age_{i}+\beta_{Age2}Age_{i}^{2}+\beta_{MS}MS_{i}+\beta_{Rural}Rural_{i}\\ & +\beta_{HHsize}HHsize_{i}+\beta_{Min}Male*\;PCHH\;income+\beta_{Fin}Female\\ & *\;PCHH\;income+\beta_{Gender}Gender_{i}+\beta_{Edu}Education_{i}\\ & +\beta_{SK}Smoke_{i}+\beta_{Alc}Alcohol_{i}+\mu_{i}+e_{i} \end{array}$$

The coefficient estimates of the UQR model specified in Equation (4) were obtained using RIF regressions at the quantiles of the BMI distribution [25]. The coefficient estimate for a specific variable in Equation (4), e.g., dietary knowledge, can be interpreted as the effect of a unit change of the variable on the BMI distribution, keeping all other independent variables constant.

We performed the UQR analysis using the whole study sample of all 9901 respondents and also conducted subgroup analyses to explore how the relationship between dietary knowledge, SES and BMI varied across different socio-economic subgroups. Subgroups were defined by income levels, i.e., low-income group, middle-income group and high-income group, and by education levels, i.e., low-education group (below college) and high-education group (college and above), respectively. The subgroup analyses evaluated the relationship between BMI and dietary knowledge and SES in each specific subsample, enabling us to identify the potential subgroups of respondents who had stronger associations between dietary knowledge, SES and BMI.

## 3. Results

### 3.1. Robustness Checks

To examine the robustness of our results, we used two different income variables, the household income per capita and the total household income, respectively, in the UQR model specified in Equation (4) and compared their results. Table 3 and Table A1 present the results from the two different models, which have little difference for all the variables, including dietary knowledge and the income variables, attributable to the nice property of UQR. Further, we used Quartic and Gaussian kernels to re-estimate Equation (4) that originally used the Epanechnikov kernel. The empirical results show that the results vary only minimally based on the function of the different kernel density functions used in the estimation of Equation (4).

### 3.2. Empirical Results

#### 3.2.1. Analysis Using the Whole Study Sample

In this analysis, we used the whole study sample of all 9901 respondents. The estimated coefficients of Equation (4) obtained using UQR and the OLS are presented in Table 3. When using the whole study sample, dietary knowledge did not have significant effects on BMI. Chinese females had statistically significant lower BMI than males. However, this relationship was not consistent across the whole BMI distribution, which was stronger at the 20th (−3.04) and 40th (−3.14) quantiles, weaker at others, and not statistically different at the 90th quantile. Age and age squared were significantly associated with BMI at all percentiles [36,40]. The coefficient estimates of age showed a decreasing trend as BMI quantiles increased. Specifically, after reaching the age of 50, adult BMI began to decline with age. In addition, the coefficient estimates did not vary largely across quantiles, suggesting an overall uniform effect of age across the BMI distribution.

Income was significantly related to higher BMI at almost all percentiles of the BMI distribution. For both females and males, as income level increased, the marginal effects of income on BMI showed a decreasing trend. However, there was a significant gender difference in the effect of income on BMI. For males, income was significantly related to higher BMI at most percentiles of the BMI distribution, while for females, the estimated coefficients became negative and statistically insignificant for the mean effect and some quantiles (e.g., the 10th, 30th, 90th quantiles).

Marital status was not significant for BMI at all quantiles of the BMI distribution although the OLS estimates showed that married individuals had higher mean BMI than unmarried individuals. Rural residency was not significantly associated with BMI at almost all quantiles, and household size had no significant relationship with BMI at all quantiles.

Education had a significant and inverse relationship with BMI at all quantiles, and the effects of education on BMI became greater at higher BMI quantiles. Smoking was significantly and inversely associated with BMI across the whole BMI distribution. Alcohol intake had a significant association with BMI almost at all BMI quantiles except for the lower levels (e.g., 10%).

#### 3.2.2. Subgroup Analysis by Income Levels

Table 4 presents the results from the subgroup analysis by income levels, including low−income group, middle-income group, and high-income group, respectively. The results for dietary knowledge were similar to those from the analysis using the whole study sample, statistically insignificant across different income groups except for the low-income group at the 90th quantile (*p* < 10%).

The age variables remained statistically significant for BMI at most BMI quantiles in all three income groups. In the middle-income and the high-income groups, difference between males and females at the 50th quantile of BMI was significant while it was not significant in the low-income group. Similar to the results from the whole study sample, rural or urban residency and marital status had no significant effects on BMI at all quantiles in all three groups, except that marital status was significant at the 90th BMI quantile in the low-income group. Smoking had significant associations with low-income and high-income obese respondents, while for the middle-income group, it was significant only at the 10th BMI quantile. Alcohol intake had a significant association with BMI at the 10th to 50th quantiles in the middle-income group while it was significant at the 40th to 80th quantiles of BMI in both middle-income and high-income groups.

#### 3.2.3. Subgroup Analysis by Education Levels

We conducted subgroup analysis using the low-education group (below college) and the high-education group (college and above), respectively. Results in Table 5 show that dietary knowledge had no statistically significant effect on the full distribution of BMI in the low-education and the high-education groups. Age remained to be a highly significant factor for BMI at all quantiles except for the 90th percentile. Marital status, household size, and rural or urban residency did not have significant associations with BMI across the distribution of BMI, except that urban residence had a significantly negative association with BMI at the 90th percentile for respondents in the high-education group. This indicates that higher education (college and above) and rural residency were associated with a higher BMI. With a 1% increase in income, the 10th, 30th, and 50th quantifiers of BMI increased by 0.30, 0.25, and 0.19 among low-educational males, respectively. However, income had no statistically significant effects on BMI for males in the high-education group. In contrast, income had no significant effect on BMI in the female low-education group, but the BMI for females in the high-education group decreased as income increased. Smoking and alcohol intake exerted different effects on BMI in different groups. The impact of smoking on BMI was greater in the low-education group than in the high-education group. Further, education had a significant effect on BMI for females, but was not significant for males (data not shown).

## 4. Discussion

Improving dietary knowledge may potentially control the rising prevalence of obesity. Using the large-scale CHNS data from 2015, we carried out a comprehensive investigation of the complex relationship between dietary knowledge, SES, and BMI among Chinese adults. We employed the UQR technique and examined how the relationship between dietary knowledge, SES, and BMI varied across the entire BMI distribution, and across different socioeconomic subgroups, categorized by income and education levels, respectively. Results for dietary knowledge remained similar in both subgroup analyses; and generally, more factors showed heterogeneous results across the different education groups than across the different income groups. The study found that dietary knowledge had no statistically significant effect on BMI across the BMI distribution, confirming previous findings [17]. One of the possible reasons is that dietary knowledge of Chinese people may be unsystematic and lacks practical guidelines [33]. Thus, the dietary knowledge has not been implemented to help them develop healthy behaviors leading to reduced risk for obesity. Healthy behavior is underpinned by a number of environmental and intra-individual factors, including accurate knowledge and motivation. Dietary knowledge may not necessarily lead to changes of dietary behaviors due to lack of motivation [41]. People might become motivated to lose weight if they believe healthy eating and weight loss will improve health, although some individuals may still fail in practicing necessary measures to control and prevent obesity because of certain environmental and intra-individual factors. It is important for nutrition promoters and educators to understand the interplay between dietary information processing, motivational factors and changes in dietary behaviors so that more effective and practical programs and guidelines can be developed.

Consistent with the literature, we found Chinese females tended to have statistically significant lower BMI than males [21]. In addition, income was significant associated with higher BMI across the BMI distribution; and there was a major gender difference in the effect of income on the BMI distribution. For males, income was significantly associated with a higher BMI, while for females, income had a negative effect. Results from subgroup analyses show that for the middle-income group, income increase had a greater impact on the 50th quantile of BMI in males, but it was not significant for females. Our findings are in contrast with previous results that middle-class individuals were more likely to be overweight and obese, without significant difference between females and males in the mean effect [42]. A possible reason is that the relationship between obesity and income may gradually change with economic growth. China has experienced expansive economic growth in the recent decades, and social structures also have undergone dramatic changes, potentially leading to changes in the relationship between social factors and obesity.

This study confirms previous findings that Chinese middle-class may suffer high risk for overweight and obesity [43]. Income has a negative relationship with BMI in most developed countries, but it is the opposite in developing countries [44]. There is enormous heterogeneity in the trend and the timing of obesity in developing countries [45]. Research shows that obesity was most prevalent in the population groups with the highest poverty rates and the least education in the US [46]. However, most results for developing countries indicate a stable positive correlation between SES and obesity [47]. The findings for China are consistent with the results from previous studies on obesity in developing countries, revealing a positive relationship between income and obesity [47,48]. From the viewpoint of economic development, there is an inflection point in the relationship between SES and obesity. As the GDP of developing countries increases, the positive relationship between SES and obesity becomes negative [48]. Hence, it is possible that with the economic development in China, the burden of obesity was gradually being transferred to the low-income and middle-income groups.

Our study found that higher education was significantly associated with reduced obesity risk, and there was a major gender difference in this relationship. As income increased, males without a college education were prone to obesity, while income had no significant effect on BMI for males with college (and above) education. In contrast, income increases had no significant effect on BMI for females without a college education, but BMI was generally lower for females with at least a college education. A possible explanation might be that women with higher education are more likely to have a higher SES, more health knowledge, and better access to more healthy resources, and consequently live healthier lifestyles [49]. Another possible reason is that females in China might particularly have attached great importance to their body image. Influenced by both Asian and Western cultures, weight control behavior and being thin is now prevalent among female students in Chinese universities, indicating that women with higher education may have cultural expectations for their body image and are less likely to become obese [50].

Consistent with previous studies, we found drinking and smoking were significantly associated with BMI. Cigarette smoking was negatively associated with BMI across the BMI distribution while alcohol intake had a positive relationship with BMI at most percentiles of BMI (30th percentile and higher). The negative relationship between smoking and BMI was well recognized [36,37,40]. A possible reason was that smoking increased physical metabolism and reduced consumption of sweet food [51]. Previous findings show that household size had a positive relationship with BMI at the lowest BMI quantiles, not confirmed by our results using the UQR models [36,37,40]. Due to the imbalance of social and economic development, SES changes may be heterogeneous across geographic regions. Our results indicate that the impact of SES on obesity showed significant geographic and urban–rural differences.

This study has some limitations. Firstly, we used cross-sectional data rather than longitudinal data. There may be temporal effects of factors on BMI over time. Prospective longitudinal studies are warranted to investigate the risk factors that may contribute to the development of obesity among Chinese adults. Secondly, according to the existing literature, BMI may be also associated with genetic factors, which this paper did not examine due to data constraints [52]. Nevertheless, this study employed the UQR approach and estimated the potential heterogenous effects of dietary knowledge and SES on BMI across the entire BMI distribution. It is among the first to provide evidence from China on the nonlinear relationship between dietary knowledge, SES, and BMI.

## 5. Conclusions

Using the large-scale CHNS data and the UQR approach, this study investigated the complex relationship between dietary knowledge, SES, and BMI among Chinese adults. Our results provide further evidence that dietary knowledge had no significant impact on BMI across the BMI distribution. There was a large degree of heterogeneity in the SES effect across the BMI distribution, as well as a major gender difference in the SES effect on BMI. Education had a significant and inverse association with BMI across the BMI distribution, which was greater at higher BMI quantiles. Income growth had a greater effect on the 50th quantile of BMI for males in the middle-income group, but was insignificant for females. Males without college educations had higher BMI as income increased, while females with high education generally had lower BMI. The findings of this study show the heterogeneous nature of the relationship between SES, gender, and obesity across the entire BMI distribution.

These findings may have public health significance. Our study suggests that it is important to consider the obesity risk of low-income people in the development of poverty alleviation strategies in China. Furthermore, we recommend that future dietary educational and promotional programs provide targeted strategies and guidelines suitable for individuals with different SES background. In addition, dietary education alone, albeit necessary, is insufficient to facilitate behavior change. In combination with dietary education, practical strategies are needed to motivate and facilitate dietary behavior changes. Moreover, individuals must also be aware of the consequences of their decisions, on whether or not to modify their dietary behaviors.

The intermediate mechanisms through which dietary knowledge affects obesity are still largely unknown and are warranted for future studies.

## Figures and Tables

**Table 1:** Dietary knowledge questions in the CHNS questionnaire and criteria.

No.	Do You Strongly Agree, Somewhat Agree, Somewhat Disagree or Strongly Disagree with this Statement, “Neutral” or “Unknown”?	True/False
Q1	Choosing a diet with a lot of fresh fruits and vegetables is good for one’s health	T
Q2	Eating a lot of sugar is good for one’s health	F
Q3	Eating a variety of foods is good for one’s health	T
Q4	Choosing a diet high in fat is good for one’s health	F
Q6	Choosing a diet with a lot of staple foods (rice and rice products and wheat and wheat products) is not good for one’s health	T
Q7	Consuming a lot of animal products daily (fish, poultry, eggs and lean meat) is good for one’s health	F
Q8	Reducing the amount of fatty meat and animal fat in the diet is good for one’s health	T
Q9	Consuming milk and dairy products is good for one’s health	T
Q10	Consuming beans and bean products is good for one’s health	T
Q11	Physical activities are good for one’s health	T
Q12	Sweaty sports or other intense physical activities are not good for one’s health	F
Q13	The heavier one’s body is, the healthier he or she is	F
Q14	Eating salty foods can cause hypertension	T
Q15	Refined grains (rice and wheat flour) contain more vitamins and materials than unrefined grains	F
Q16	Lard is healthier than vegetable oils	F
Q17	Vegetables contain more starch than staple foods (rice or wheat flour)	F
Q18	Eggs and milk are important sources of high-quality protein	T

Source: China Health and Nutrition Survey (CHNS) and Clément and Bonnefond [26].

**Table 2:** Descriptive statistics of BMI, income and other variables of the respondents.

Variable	N	Mean	Std. Dev.	Min	Max
BMI	9901	24.29	4.10	15	44
Dietary Knowledge (DK)	9901	20.24	2.94	5	28
Age	9901	50	14.74	18	94
Age Squared	9901	2731	1498	324	8836
Marital Status (unmarried = 0, married = 1)	9901	2.06	0.59	1	9
Rural (rural = 0,uburan = 1)	9901	0.43	0.49	0	1
HH size ^1^	9901	3.62	1.64	1	15
HH income ^2^	9901	81,394	117,141	0	4,528,302
PCHH income ^3^	9901	25,008	35,733	0	1,132,075
Gender (female = 0, male = 1)	9901	0.50	0.50	0	1
Education	9901	2.70	1.38	1	9
Smoke (yes = 1, no = 0)	9901	0.25	0.43	0	1
Alcohol (yes = 1, no = 0)	9901	0.08	0.27	0	1

Source: China Health and Nutrition Survey (CHNS). ^1^ HH: household size. ^2^ HH income: household income.^3^ PCHH income: household income per capita.2.2. Statistical Analysis.

**Table 3:** Ordinary least squares (OLS) and unconditional quantile regression (UQR) results (*N =* 9901 observations).

	10th	30th	50th	70th	90th	OLS
Dietary knowledge	−0.02	0.0025	−0.01	0.01	0.02	0.01
	(−1.45)	(0.18)	(−0.87)	(0.57)	(0.97)	(0.82)
Age	0.34 ***	0.27 ***	0.21 ***	0.16 ***	0.04	0.20 ***
	(12.78)	(14.61)	(11.32)	(7.52)	(1.54)	(11.30)
Age squared	−0.0031 ***	−0.0024 ***	−0.0019 ***	−0.0015 ***	−0.0005 *	−0.0019 ***
	(−12.39)	(−13.76)	(−10.72)	(−7.21)	(−2.05)	(−10.92)
Marital status	0.16	0.12	0.09	0.10	0.19	0.22 **
	(1.61)	(1.59)	(1.08)	(1.01)	(1.55)	(2.88)
Rural	0.05	0.06	0.06	−0.10	−0.38 **	−0.06
	(0.51)	(0.69)	(0.57)	(−0.85)	(−2.59)	(−0.67)
HH size ^1^	0.01	−0.05	−0.03	−0.01	−0.04	−0.04
	(0.27)	(−1.48)	(−1.04)	(−0.28)	(−0.92)	(−1.25)
Male * PCHH income ^2^	0.21 ***	0.26 ***	0.22 ***	0.13	0.0537	0.20 ***
	(3.41)	(4.82)	(3.69)	(1.77)	(0.59)	(3.59)
Female *PCHH income^2^	−0.02	−0.08	−0.13 *	−0.19 **	0.05	−0.09
	(−0.35)	(−1.48)	(−2.25)	(−2.74)	(0.60)	(−1.69)
Gender	−1.77 *	−2.85 ***	−2.73 ***	−2.36 *	0.19	−2.54 **
	(−2.09)	(−3.93)	(−3.55)	(−2.57)	(0.18)	(−3.13)
Year of education	−0.03	−0.10 **	−0.15 ***	−0.16 ***	−0.21 ***	−0.13 ***
	(−0.80)	(−2.74)	(−3.99)	(−3.40)	(−3.71)	(−3.63)
Smoke	−0.51 ***	−0.54 ***	−0.68 ***	−0.75 ***	−0.55 **	−0.69 ***
	(−3.80)	(−4.53)	(−4.95)	(−5.18)	(−3.21)	(−6.06)
Alcohol	0.05	0.10 **	0.12 ***	0.14 ***	0.11 *	0.092 **
	(1.31)	(3.24)	(3.49)	(3.46)	(2.26)	(3.01)
Province1	−0.42 *	−0.240	−0.12	−0.08	−0.31	−0.18
	(−2.12)	(−1.28)	(−0.56)	(−0.28)	(−0.85)	(−0.86)
Province2	−0.29	−0.43 *	−0.48 *	−0.97 ***	−0.91 *	−0.43 *
	(−1.48)	(−2.18)	(−2.12)	(−3.43)	(−2.51)	(−2.05)
Province3	−0.51 **	−0.52 **	−0.78 ***	−1.21 ***	−1.03 **	−0.90 ***
	(−2.73)	(−2.90)	(−3.81)	(−4.76)	(−3.24)	(−4.65)
Province4	−0.70 **	−1.05 ***	−1.01 ***	−1.50 ***	−1.28 ***	−1.12 ***
	(−3.22)	(−5.30)	(−4.56)	(−5.51)	(−3.76)	(−5.39)
Province5	0.076	0.07	0.29	0.19	0.16	0.10
	(0.44)	(0.38)	(1.32)	(0.64)	(0.41)	(0.45)
Province6	−0.59 **	−0.51 *	−0.51*	−0.67 *	−0.19	−0.51 *
	(−2.63)	(−2.48)	(−2.16)	(−2.26)	(−0.48)	(−2.32)
Province7	−1.11 ***	−1.28 ***	−1.46 ***	−1.98 ***	−1.68 ***	−1.42 ***
	(−4.75)	(−6.22)	(−6.45)	(−7.25)	(−4.90)	(−6.67)
Province8	−0.78 ***	−1.07 ***	−1.21 ***	−1.614 ***	−1.64 ***	−1.40 ***
	(−3.63)	(−5.36)	(−5.48)	(−5.98)	(−4.92)	(−6.74)
Province9	−2.18 ***	−2.067 ***	−2.34 ***	−2.83 ***	−2.31 ***	−2.45 ***
	(−8.46)	(−9.91)	(−10.69)	(−10.87)	(−7.26)	(−11.63)
Province10	−1.46 ***	−1.49 ***	−1.56 ***	−1.95 ***	−1.63 ***	−1.734 ***
	(−5.86)	(−7.13)	(−6.93)	(−7.23)	(−4.82)	(−8.19)
Province11	−0.92 ***	−0.76 ***	−0.80 ***	−1.17 ***	−0.89 *	−0.98 ***
	(−3.87)	(−3.62)	(−3.37)	(−4.01)	(−2.40)	(−4.46)
Constant	12.22 ***	16.80 ***	21.26 ***	24.84 ***	28.76 ***	20.87 ***
	(11.92)	(21.06)	(25.70)	(25.57)	(24.49)	(25.47)

Standard Errors are in Parenthese.* Parameters are statistically different from 0 at a 10% probability level. ** Parameters are statistically different from 0 at a 5% probability level. *** Parameters are statistically different from 0 at a 1% probability level. ^1^ HH: household size. ^2^ PCHH income: household income per capita.

**Table 4:** Estimated relationship of dietary knowledge and socioeconomic status (SES) on body mass index (BMI) by income group.

	Low-Income			Middle-Income			High-Income		
	10th	50th	90th	10th	50th	90th	10th	50th	90th
Dietary knowledge	−0.03	0.05	0.13 *	−0.02	−0.05	−0.05	−0.02	−0.04	−0.05
	(−0.82)	(1.03)	(2.06)	(−0.38)	(−1.28)	(−0.70)	(−0.62)	(−1.13)	(−0.88)
Age	0.26 ***	0.24 ***	−0.01	0.53 ***	0.20 ***	0.00001	0.377 ***	0.27 ***	0.10
	(4.19)	(3.40)	(−0.16)	(6.02)	(4.53)	(0.00)	(6.83)	(6.33)	(1.75)
Age Squared	−0.0026 ***	−0.0024 ***	−0.0002	−0.0048 ***	−0.0017 ***	−0.0003	−0.0035 ***	−0.0023 ***	−0.0009
	(−4.28)	(−3.64)	(−0.33)	(−5.81)	(−4.07)	(−0.41)	(−6.64)	(−5.73)	(−1.63)
Marital status	0.18	0.12	0.71 *	−0.25	0.09	0.40	0.20	−0.06	0.19
	(1.17)	(0.47)	(2.08)	(−0.64)	(0.39)	(0.99)	(0.70)	(−0.31)	(0.59)
Rural	−0.29	0.25	0.18	−0.22	−0.21	−0.57	0.08	−0.30	−1.00 **
	(−1.04)	(0.63)	(0.42)	(−0.68)	(−0.86)	(−1.32)	(0.39)	(−1.35)	(−3.16)
HH size ^1^	−0.01	−0.14	−0.09	−0.15	−0.10	−0.18	−0.05	0.02	0.15
	(−0.11)	(−1.31)	(−0.86)	(−1.08)	(−1.18)	(−1.15)	(−0.65)	(0.20)	(1.23)
Male * PCHH income ^2^	−0.19	−0.23	−0.32	0.27	2.73 *	−0.82	0.23	−2.64 *	−1.34
	(−1.68)	(−1.09)	(−1.23)	(0.16)	(2.13)	(−0.37)	(0.23)	(−2.23)	(−0.78)
Female * PCHH income ^2^	−0.02	−0.13	0.06	2.70	−0.92	−1.95	−1.38	−1.37	−1.70
	(−0.14)	(−0.65)	(0.26)	(1.49)	(−0.75)	(−0.80)	(−1.22)	(−1.17)	(−1.11)
Gender	1.27	0.95	4.38	27.58	−39.29 *	−12.87	−17.65	15.35	−3.84
	(0.82)	(0.36)	(1.43)	(1.02)	(−2.05)	(−0.36)	(−1.02)	(0.82)	(−0.15)
Year of Education	0.11	−0.19	0.07	−0.17	−0.12	−0.58 **	−0.03	−0.15	−0.09
	(1.02)	(−1.22)	(0.39)	(−1.22)	(−1.23)	(−3.23)	(−0.38)	(−1.91)	(−0.78)
Smoke	−0.32	−1.10 *	−0.78 *	−1.08 *	−0.54	−0.55	−0.34	−0.96 **	−0.89 *
	(−1.21)	(−1.98)	(−2.06)	(−2.51)	(−1.76)	(−1.07)	(−1.26)	(−3.27)	(−2.09)
Alcohol	0.06	0.13	0.03	0.21 *	0.19 *	0.15	−0.02	0.22 **	0.21
	(0.61)	(1.02)	(0.21)	(2.15)	(2.34)	(1.12)	(−0.33)	(2.94)	(1.91)
Fixed effect	yes								
N	1500	1500	1500	1897	1897	1897	1945	1945	1945

Standard Errors are in Parenthese.* Parameters statistically different from 0 at a 10% probability level. ** Parameters are statistically different from 0 at a 5% probability level. *** Parameters are statistically different from 0 at a 1% probability level. Coefficients for Region Fixed−Effects are omitted for brevity. ^1^ HH size: household size. ^2^ PCHH income: household income per capita.

**Table 5:** Estimated relationship between dietary knowledge and SES on BMI by education group.

Variable	Low					High				
	10th	30th	50th	70th	90th	10th	30th	50th	70th	90th
Dietary knowledge	−0.03	−0.0028	−0.01	0.002	0.02	−0.02	0.0039	0.0046	0.02	0.07
	(−1.30)	(−0.17)	(−0.56)	(0.09)	(0.79)	(−0.48)	(0.12)	(0.14)	(0.59)	(1.65)
age	0.38 ***	0.27 ***	0.21 ***	0.15***	0.04	0.30 ***	0.30 ***	0.22***	0.20 ***	0.01
	(9.96)	(11.70)	(8.68)	(6.08)	(1.32)	(6.59)	(7.63)	(6.18)	(4.67)	(0.30)
Age Squared	−0.0034 ***	−0.0024 ***	−0.0019 ***	−0.0014***	−0.0005	−0.0026 ***	−0.0026 ***	−0.0019***	−0.0018 ***	−0.0002
	(−9.90)	(−11.25)	(−8.46)	(−5.88)	(−1.71)	(−6.10)	(−6.90)	(−5.39)	(−4.28)	(−0.61)
Marital status	0.17	0.06	0.10	0.09	0.16	0.12	0.26	0.14	0.23	0.22
	(1.30)	(0.67)	(0.97)	(0.80)	(1.11)	(0.77)	(1.50)	(0.78)	(0.98)	(1.02)
Rural	0.16	0.08	0.10	−0.02	−0.12	−0.06	−0.07	−0.23	−0.44	−0.84 ***
	(1.12)	(0.78)	(0.81)	(−0.11)	(−0.70)	(−0.30)	(−0.35)	(−1.24)	(−1.85)	(−3.36)
HH size^1^	−0.0080	−0.06	−0.05	0.01	−0.04	−0.06	−0.06	0.02	0.0090	−0.02
	(−0.16)	(−1.79)	(−1.34)	(0.26)	(−0.79)	(−0.60)	(−0.73)	(0.21)	(0.10)	(−0.24)
Male * PCHH income ^2^	0.30 ***	0.25 ***	0.19 **	0.10	0.06	0.22	0.04	−0.04	−0.28	−0.43
	(3.80)	(3.90)	(2.75)	(1.29)	(0.59)	(1.53)	(0.30)	(−0.28)	(−1.37)	(−1.77)
Female * PCHH income ^2^	0.02	−0.02	−0.07	−0.12	0.08	−0.34 *	−0.19	−0.29 *	−0.36 *	−0.11
	(0.24)	(−0.28)	(−1.01)	(−1.55)	(0.91)	(−2.23)	(−1.14)	(−2.02)	(−2.00)	(−0.65)
Gender	−2.78 *	−2.78 **	−2.48 *	−2.01	0.21	−5.29 *	−0.93	−1.10	0.90	4.53
	(−2.32)	(−2.99)	(−2.42)	(−1.77)	(0.16)	(−2.25)	(−0.40)	(−0.49)	(0.30)	(1.39)
Smoke	−0.56 **	−0.51 ***	−0.78 ***	−0.78 ***	−0.64 ***	−0.51 *	−0.48 *	−0.39	−0.33	0.23
	(−3.05)	(−3.53)	(−4.49)	(−4.97)	(−3.49)	(−2.11)	(−1.97)	(−1.49)	(−0.95)	(0.60)
Alcohol	0.06	0.06	0.11 **	0.13 **	0.11	0.07	0.19 ***	0.10	0.12	0.05
	(1.10)	(1.55)	(2.66)	(2.83)	(1.88)	(1.33)	(3.54)	(1.74)	(1.62)	(0.73)
constant	11.19 ***	16.65 ***	21.09 ***	24.66 ***	28.45 ***	16.54 ***	16.09 ***	20.73 ***	23.84 ***	28.25 ***
	(7.85)	(16.95)	(19.67)	(20.96)	(19.11)	(7.68)	(7.50)	(11.02)	(10.37)	(13.03)
Fixed effect	yes									
N	6706					2305				

Standard Errors are in Parentheses.* Parameters are statistically different from 0 at a 10% probability level. ** Parameters are statistically different from 0 at a 5% probability level. *** Parameters are statistically different from 0 at a 1% probability level. Coefficients for Region Fixed-Effects are omitted for brevity. ^1^ HH size: household size. ^2^ PCHH: household income per capita.

## Appendix A

**Table A1:** Robustness check: ordinary least squares (OLS) and unconditional quantile regression (UQR) using household income as an independent variable (*N =* 9901 observations).

Variable	10th	30th	50th	70th	90th
dietary knowledge	−0.02	0.002	−0.01	0.01	0.02
	(−1.47)	(0.17)	(−0.87)	(0.57)	(0.98)
Age	0.34 ***	0.27 ***	0.21 ***	0.16 ***	0.04
	(12.75)	(14.55)	(11.28)	(7.49)	(1.54)
Age Squared	−0.0031 ***	−0.0024 ***	−0.0019 ***	−0.0015 ***	−0.0005 *
	(−12.35)	(−13.69)	(−10.67)	(−7.19)	(−2.04)
Marital status	0.15	0.11	0.08	0.09	0.19
	(1.53)	(1.49)	(0.95)	(0.89)	(1.56)
Rural	0.05	0.06	0.05	−0.11	−0.37 **
	(0.45)	(0.62)	(0.51)	(−0.89)	(−2.58)
HH size ^1^	−0.02	−0.07 *	−0.04	−0.0022	−0.05
	(−0.40)	(−2.24)	(−1.38)	(−0.06)	(−1.18)
Male * HH income ^2^	0.28 ***	0.27 ***	0.23 ***	0.12	0.05
	(4.40)	(4.97)	(3.82)	(1.68)	(0.57)
Female *HH income ^2^	−0.07	−0.09	−0.14 *	−0.18 **	0.03
	(−1.07)	(−1.56)	(−2.49)	(−2.64)	(0.39)
Gender	−3.22 ***	−3.40 ***	−3.40 ***	−2.598 *	0.02
	(−3.40)	(−4.15)	(−3.88)	(−2.47)	(0.02)
Year of Education	−0.03	−0.10 **	−0.15 ***	−0.16 ***	−0.21 ***
	(−0.83)	(−2.76)	(−3.99)	(−3.41)	(−3.69)
Smoke	−0.52 ***	−0.55 ***	−0.69 ***	−0.76 ***	−0.55 **
	(−3.83)	(−4.60)	(−5.01)	(−5.25)	(−3.22)
Alcohol	0.04	0.10 **	0.11 ***	0.13 ***	0.11 *
	(1.26)	(3.15)	(3.41)	(3.40)	(2.26)
Province 1	−0.42 *	−0.24	−0.12	−0.078	−0.31
	(−2.13)	(−1.28)	(−0.56)	(−0.28)	(−0.85)
Province 2	−0.29	−0.42 *	−0.47 *	−0.97 ***	−0.91 *
	(−1.46)	(−2.15)	(−2.10)	(−3.42)	(−2.51)
Province 3	−0.52 **	−0.52 **	−0.78 ***	−1.21 ***	−1.03 **
	(−2.76)	(−2.91)	(−3.81)	(−4.76)	(−3.23)
Province 4	−0.70 **	−1.05 ***	−1.01 ***	−1.49 ***	−1.28 ***
	(−3.24)	(−5.29)	(−4.55)	(−5.50)	(−3.75)
Province 5	0.08	0.08	0.29	0.19	0.16
	(0.46)	(0.41)	(1.34)	(0.65)	(0.41)
Province 6	−0.58 **	−0.51 *	−0.51 *	−0.67 *	−0.19
	(−2.60)	(−2.43)	(−2.14)	(−2.24)	(−0.48)
Province 7	−1.10 ***	−1.28 ***	−1.46 ***	−1.98 ***	−1.68 ***
	(−4.72)	(−6.18)	(−6.42)	(−7.23)	(−4.90)
Province 8	−0.78 ***	−1.06 ***	−1.21 ***	−1.61 ***	−1.64 ***
	(−3.63)	(−5.34)	(−5.47)	(−5.97)	(−4.93)
Province 9	−2.16 ***	−2.06 ***	−2.34 ***	−2.83 ***	−2.31 ***
	(−8.42)	(−9.87)	(−10.67)	(−10.86)	(−7.26)
Province 10	−1.46 ***	−1.49 ***	−1.56 ***	−1.95 ***	−1.63 ***
	(−5.85)	(−7.13)	(−6.94)	(−7.24)	(−4.82)
Province 11	−0.920 ***	−0.758 ***	−0.79 ***	−1.16 ***	−0.89 *
	(−3.86)	(−3.61)	(−3.35)	(−3.99)	(−2.41)
Constant	12.88 ***	17.08 ***	21.66 ***	25.02 ***	28.92 ***
	(12.25)	(20.58)	(25.06)	(24.41)	(23.38)

Standard Errors in Parenthesis.* Parameters statistically different from 0 at the 10% probability level. ** Parameters statistically different from 0 at the 5% probability level. *** Parameters statistically different from 0 at the 1% probability level. ^1^ HH size: household size. ^2^ PCHH income: household income.
